# Study on Damage and Tensile Properties of Thermal-Treated Granite Under Different Thermal Shock Conditions

**DOI:** 10.3390/ma19071404

**Published:** 2026-04-01

**Authors:** Kun Li, Xiaoming Zhao, Haoyu Wang, Dongjie Li, Donghong Dang, Yan Xi

**Affiliations:** 1 School of Geosciences and Technology, Southwest Petroleum University, Chengdu 610500, China; cy5_likun@petrochina.com.cn (K.L.); zhxim98@163.com (X.Z.); 2 No. 4 Oil Production Plant, PetroChina Huabei Oilfield Company, Langfang 065000, China; 3 Sichuan Key Laboratory of Natural Gas Geology, Chengdu 610500, China; 4 College of Architecture and Civil Engineering, Beijing University of Technology, Beijing 100124, China; wanghaoyu2122@163.com; 5 Development Department, PetroChina Huabei Oilfield Company, Renqiu 062552, China; cyy_lidj@petrochina.com.cn; 6 Engineering Technology Research Institute, PetroChina Bohai Drilling Engineering Co., Ltd., Tianjin 300457, China; dangdonghong@cnpc.com.cn

**Keywords:** hot dry rock, thermal shock damage, Brazilian splitting test, section roughness

## Abstract

With the development of deep geothermal resources, including hot dry rock, the issues of low rock-breaking efficiency and wellbore instability encountered when drilling into high-temperature granite reservoirs have become increasingly prominent. The study aims to elucidate the physical degradation and fracture failure mechanisms of granite exposed to high temperatures and thermal shock. The mineral composition and microstructure of granite were analyzed by X-ray diffraction (XRD) combined with field emission scanning electron microscopy (FE-SEM). Systematic experiments were conducted to investigate the thermal damage mechanisms and mechanical properties of thermal-treated (25 °C to 600 °C) granite under different cooling conditions (natural cooling, water cooling, LN_2_ cooling). The experimental results show that the physical parameters of granite exhibit significant path dependence on temperature and cooling rate. When the temperature exceeds 400 °C, the rock undergoes pronounced nonlinear volumetric expansion and a sharp increase in porosity, with P-wave velocity decaying exponentially as the temperature rises. Mechanical tests reveal that high temperature considerably weakens the rock tensile strength. For granite at 600 °C, the maximum reduction in strength reaches 80.79%, and faster cooling leads to greater strength degradation. Additionally, 3D morphology analysis indicates that the section roughness of granite increases exponentially with temperature, where the arithmetic mean height *S_a_* more comprehensively reflects the overall characteristics of surface morphology and demonstrates the strongest ability for characterizing strength. These findings provide a theoretical basis for the efficient volumetric fracturing and rapid drilling technologies applicable to hot dry rock.

## 1. Introduction

In the context of profound transformation in the global energy structure, deep geothermal resources, particularly hot dry rock, are considered a vital pillar of the future clean energy system due to their vast reserves, widespread distribution, and extremely low carbon emissions [1,2,3,4]. While deep granite formations across several key regions in China have been confirmed to possess substantial geothermal development potential, their commercialization has always been restricted by bottlenecks in drilling and extraction technologies [5]. Typical hot dry rock reservoirs in China are mostly located at depths of 3000 to 5000 m, with temperatures generally ranging between 150 and 250 °C. In high-temperature anomaly zones such as the Gonghe Basin in Qinghai, the bottom-hole temperature at a 3700 m depth has been measured to exceed 236 °C [6]. The coupled effects of high temperature and high geostress lead to profound thermal softening, thermal fracturing, and rheological behaviors in rocks, which pose various engineering challenges for conventional drilling technologies, including low rock-breaking efficiency, abnormal drill bit wear, and frequent wellbore instability [7,8]. Fundamentally, these problems stem from the current insufficient understanding of the physical and mechanical behaviors of rocks under high-temperature and high-pressure conditions, alongside the absence of theoretical models that explain the rock-breaking mechanisms influenced by coupled thermal-mechanical effects. Hence, revealing the damage evolution patterns of rocks in high-temperature settings is not only a theoretical inquiry at the mechanical level but also an urgent necessity to overcome the technical bottleneck related to economically and efficiently developing hot dry rock.

In high-temperature environments, rock materials undergo thermal cracking, resulting in significant alterations in their physical and mechanical properties, such as pore structure, deformation characteristics, and mechanical strength. In recent years, scholars worldwide have systematically explored the physical and mechanical behaviors of rocks under high-temperature conditions [9,10,11,12]. Zhang et al. examined how changes in mineral composition, internal structure, and moisture content of thermal-treated rocks influence their physical and mechanical properties, summarizing the temperature-dependent process of physical property variations into three distinct phases [13]. Li et al. revealed that the effect of quartz on the mechanical strength of granite is the most significant under high temperature and low pore pressure conditions, while its effect on the elastic modulus is obvious under high temperature and high pore pressure. Then feldspar has the greatest impact on the elastic modulus of granite under high temperature and low pore pressure [14]. Khan et al. comparatively investigated the physical and mechanical properties of thermal-treated granites with different textures, noting that as the temperature rises, pre-existing cracks in major minerals such as feldspar and quartz progressively propagate, accompanied by the formation of new cracks induced by high thermal stress, both of which affect the rock compressive strength [15]. Fan et al. explored the uniaxial compression characteristics and microstructural evolution of thermal-treated granite under cyclic loading, finding that with the increase in the number of thermal cycles, both the porosity and maximum strain of the granite increased, while the Young’s modulus decreased [16].

Taken collectively, current studies predominantly focus on characterizing the compressive properties of rocks, while in-depth investigation into their tensile properties remains relatively scarce. It is noteworthy that the tensile strength of rock materials is often merely 1/10 to 1/20 of their compressive strength, indicating that even under a uniaxial compressive stress field, significant local tensile stress failures can occur in the rocks due to stress concentration effects [17,18].

When subjected to heat, rocks experience thermal stress that accelerates the buildup of tensile stress, which in turn exacerbates the degradation of their strength and deformation properties. In recent years, research into the rock tensile properties has been gradually undertaken [19,20,21]. Kumari et al. explored how temperature influences the tensile strength of granite, alongside the corresponding microstructural alterations arising from thermal treatment. They verified the initiation and propagation of cracks during their experiments through acoustic emission (AE) technology and scanning electron microscopy (SEM) [22]. Wang et al. examined the changes in thermal-induced cracks and tensile strength of granite subjected to rapid heating, and analyzed the tensile behavior of granite under rapid heating at varying temperatures by employing the digital image correlation (DIC) technology [23]. Zhang et al. further assessed the tensile deformation process of high-temperature granite with strain gauges, observing that the tensile stress–strain curves transition from linear to nonlinear as the temperature rises, the axial tensile strain at the failure point increases, while the tensile strength and tensile elastic modulus diminish progressively with the rising temperature [24]. Through uniaxial compression and tensile tests on rocks of varying lithologies, Tan et al. established different unloading points prior to the peak stress to differentiate between elastic energy and dissipated energy, and analyzed the damage evolution process of three rock types during tensile loading from an energy perspective [25]. While researchers are increasingly focusing on the tensile strength of rocks, existing studies still insufficiently deal with the influence of cooling methods on this property and the characterization of failure modes. Moreover, research concerning the cooling process primarily emphasizes its impact on the uniaxial compressive performance of rocks. Considering that rock tensile strength is a key metric for evaluating the deep underground engineering stability and optimizing the drilling tool parameters, systematic experiments are urgently needed to further uncover its evolution mechanism under varying temperature conditions.

Hence, utilizing standard granite discs as the research object, this study aims to elucidate the physical degradation and fracture failure mechanisms of granite exposed to high temperatures and thermal shock. A combination of X-ray diffraction (XRD) and field emission scanning electron microscopy (FE-SEM) was employed to analyze the mineral composition and microstructure of granite. Systematic experiments were conducted to investigate the thermal damage mechanisms and mechanical properties of thermal-treated (25 °C to 600 °C) granite that were subjected to natural cooling, water cooling, and LN_2_ cooling. Furthermore, by applying three-dimensional (3D) scanning technology, fracture surfaces were reconstructed, three parameters for evaluating fracture surface roughness were defined, and a quantitative model correlating the thermal damage factor with the tensile strength was developed. The findings of this study provide theoretical support for efficient volumetric fracturing and rapid drilling technologies that are applicable to hot dry rock.

## 2. Materials and Methods

### 2.1. Sample Preparation

**Figure 1:**
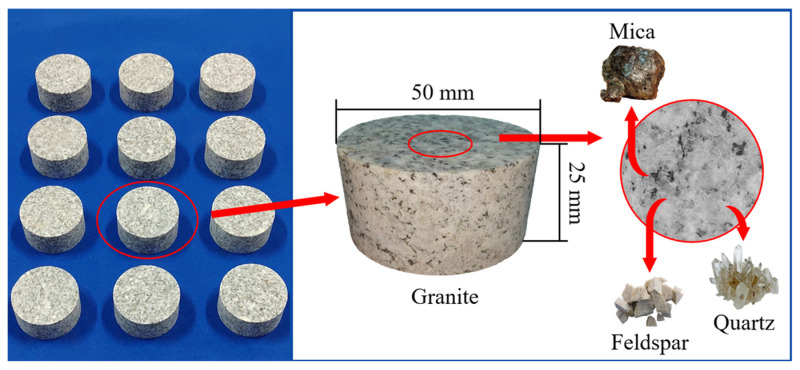
Selection and size of granite specimens.

Figure 1 shows the processed standard granite disc specimens, along with the correlation between their microscopic mineral composition and macroscopic physical dimensions. The left image displays the granite specimens with a diameter of 50 mm and a height of 25 mm, which have been polished and screened for testing purposes, demonstrating the reproducibility and statistical significance of the study [26]. According to the rock dynamic testing methods recommended by prior researchers [27], selecting specimens of this size for experiments fulfills the assumption of stress uniformity [28,29]. The right image, presented in an enlarged view, reveals the heterogeneous multiphase composite structure of the granite, clearly identifying its primary components as mica, feldspar, and quartz.

### 2.2. Component Analysis

**Figure 2:**
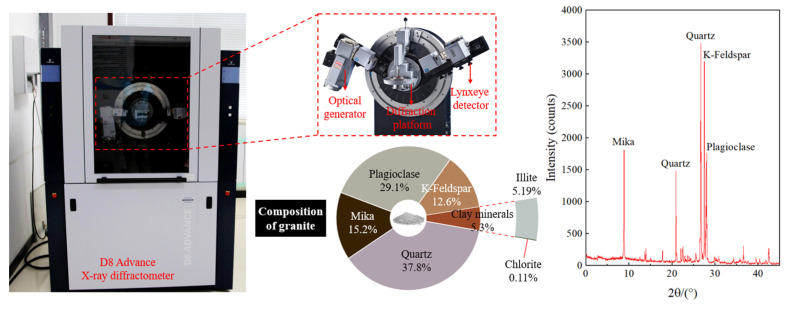
X-ray diffractometer and component analysis of rock.

Multi-scale characterization of granite, from macroscopic specimens to microscopic components, usually uncovers the intrinsic relationship of its physical and mechanical properties with its inherent mineral composition and structural features [30]. Consequently, further investigation into the microscopic composition of granite is warranted. The mineral composition of granite was examined using the D8 Advance XRD system (Bruker, Karlsruhe, Germany), as shown in Figure 2, which is a core instrument currently applied in disciplines such as materials science and geology for analyzing crystal structures and phase compositions. The goniometer platform shown in the figure constitutes its core testing unit, with key components including an optical generator, a diffraction platform, and a Lynxeye detector. This configuration enables high-precision angle control and high-intensity signal acquisition.

Figure 2 also presents the results of mineral composition analysis for a representative granite specimen based on XRD data. The granite composition reveals typical heterogeneous characteristics, with the primary minerals being quartz (37.8%) and plagioclase (29.1%), which together account for over 2/3 of the whole rock, reflecting the typical feature of an acidic intrusive rock. The secondary minerals include mica (15.2%), K-feldspar (12.6%), and clay minerals (5.3%). Among the clay minerals, illite (5.19%) and chlorite (0.11%) are present in such small amounts that they are classified as secondary or trace mineral phases. From the mineral proportion perspective, the high abundance of quartz and plagioclase dominates the construction of the rock’s rigid framework, while the presence of mica and clay minerals may considerably influence the rock’s hydro-physical properties. This provides a key mineralogical reference for the subsequent inversion of mechanical parameters for granite and the evaluation of its physical and mechanical characteristics.

### 2.3. Heating and Cooling Treatments

**Figure 3:**
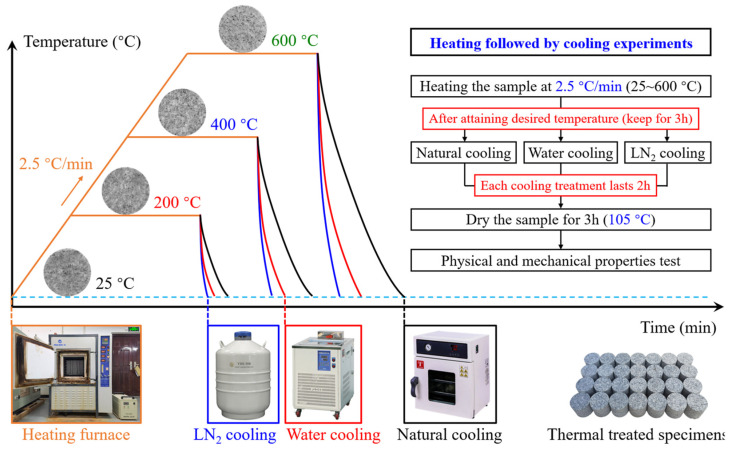
Heating and cooling treatments of granite.

The heating treatment processes of the rock specimens under different temperature conditions are illustrated in Figure 3, along with the experimental setup and the corresponding experimental procedures. Three desired temperature levels of 200 °C, 400 °C, and 600 °C were established, with specimens at 25 °C serving as controls. For each desired temperature, three specimens were selected for each of the three cooling methods to investigate the coupled effects of heating and cooling treatments on the physical and mechanical properties of granite.

During the experiments, an MXQ1200-50 heating furnace (Weixing, Shanghai, China) was utilized for precise temperature-controlled heating. The specimens were heated from 25 °C to 200/400/600 °C at a 2.5 °C/min rate and kept at the desired temperatures for 3 h to ensure thorough thermal treatment [26]. Subsequently, the specimens were divided into three groups and cooled by three distinct methods: natural cooling (in a constant temperature and humidity chamber), water cooling (in a water bath), and LN_2_ cooling (using liquid nitrogen). Each cooling treatment lasted for 2 h [31]. Following the cooling process, all specimens were dried at 105 °C for 3 h to eliminate the impact of moisture content fluctuations on the test results. Finally, all specimens were naturally cooled to room temperature inside a heating furnace, in preparation for further testing.

**Figure 4:**
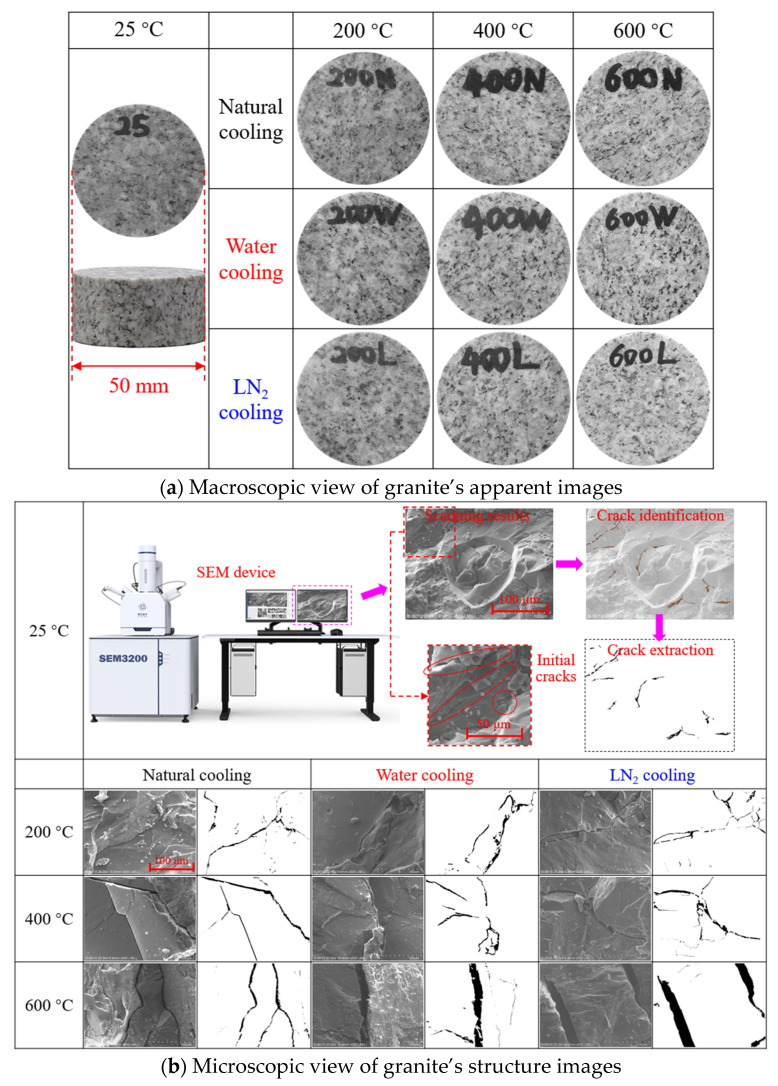
Specimen observation after thermal and cooling treatments.

To further investigate the most direct impact of heating and cooling treatments on the granite specimens, the treated specimens were observed from both macroscopic and microscopic perspectives, with specific findings depicted in Figure 4. In particular, Figure 4a presents the macroscopic morphological characteristics of granite specimens that are subjected to varying temperatures and cooling techniques. It is evident that as the temperature rises, the specimens treated by all three cooling methods exhibit a phenomenon of surface rock discoloration (lightening), most noticeably for the specimens heated to 600 °C. This phenomenon is predominantly associated with the physicochemical processes, such as thermal-induced mineral phase transformation, microcrack development, and surface oxidation [32].

Given the inability to observe the development and evolution of microcracks on the specimen surface at the macroscopic level, this study employed the argon-ion polishing FE-SEM to characterize the microscopic morphology and crack distribution characteristics of the specimens, as depicted in Figure 4b. For a more intuitive visualization of the crack morphology, the main cracks in the specimens were extracted through binarization processing. When combined with the FE-SEM findings, it becomes evident that the granite is relatively dense at room temperature, with fewer microcracks and micropores. In contrast, for granite specimens exposed to heating and cooling treatments, the number of microcracks and micropores increased significantly, with both the microcrack length and width increasing notably and the crack propagation paths turning more intricate. Among the specimens subjected to various cooling treatments, the crack densities of water-cooled and LN_2_-cooled specimens at high temperatures were markedly higher compared to those of naturally cooled specimens, suggesting that rapid cooling exacerbates the initiation and propagation of cracks induced by thermal stress. The naturally cooled specimens exhibit fewer cracks at 200 °C, whereas the water-cooled and LN_2_-cooled specimens display noticeable cracks at the same temperature, highlighting the substantial influence of cooling rate on the crack formation. Moreover, the primary crack width in the 600 °C specimen cooled with LN_2_ increased significantly, reflecting the concentration of internal residual stress within the material due to thermal treatment followed by rapid cooling treatment, which results in a decrease in fracture toughness. Overall, the coupled effect of high temperature and rapid cooling aggravates the material’s embrittlement and crack propagation, which provides a microscopic basis for understanding the failure mechanism of the material under thermal shock conditions [33].

### 2.4. Physical Properties Testing

**Figure 5:**
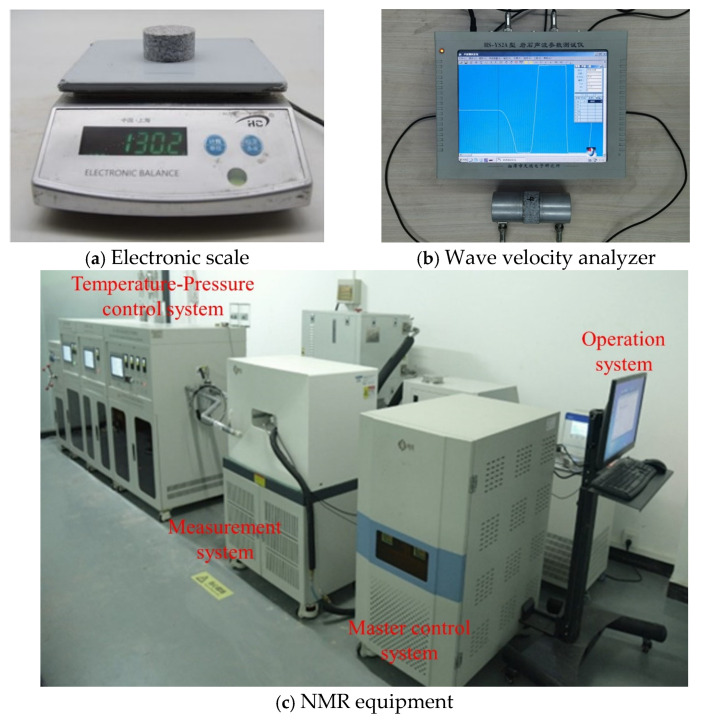
Experimental determination of physical properties of thermal-treated granite.

The mass and volume of granite specimens were measured before and after the heating and cooling treatments, respectively, to determine the density of each specimen (Figure 5a). The HS-YS2A wave velocity analyzer (Figure 5b), based on ultrasonic pulse transmission technology, was used to measure the P-wave velocity of the specimens prior to and following the heating and cooling processes. Each specimen was positioned between two testing sensors, ensuring that all three were aligned horizontally. To reduce the wave velocity attenuation, a specific amount of petroleum jelly was applied to both specimen ends. A certain force was exerted on the two sensors to guarantee their close contact with the specimen [34]. The average P-wave velocity for each group of three specimens before and after each heating and cooling treatment was computed to serve as the final measurement result, thereby ensuring the reliability of the findings.

Nuclear magnetic resonance (NMR) technology is recognized as a valuable tool for characterizing the pore size and distribution features of porous media, both before and after heating treatment. The NMR equipment utilized in this study is the MicroMR12-025V LF NMR core analysis system (NIUMAG, Suzhou, China), as shown in Figure 5c. By directly detecting the hydrogen nuclei signals in the specimens, this technique allows for the measurement of basic physical parameters such as specimen porosity and permeability. Consequently, water saturation treatment was performed on the granite specimens prior to the NMR experiments to ensure complete filling of the rock pores with liquid. Following the saturation process, the specimens were removed and wrapped with cling film after wiping off any surface free water to prevent moisture evaporation, which could potentially affect the NMR results. Subsequently, the NMR experiments were conducted, during which the laboratory temperature was strictly controlled and maintained at 25.00 ± 0.50 °C. The testing system was configured with a 12 MHz NMR frequency and probe coils measuring 60 mm in diameter, with signal acquisition achieved using the CPMG pulse sequence. The experimental parameters were set as follows: echo time (TE) = 0.1 ms, wait time (TW) = 3000 ms, echo number (NECH) = 8000, and number of scans (NS) = 128, to ensure the accuracy and reliability of the test data [35].

### 2.5. Brazilian Splitting Test

**Figure 6:**
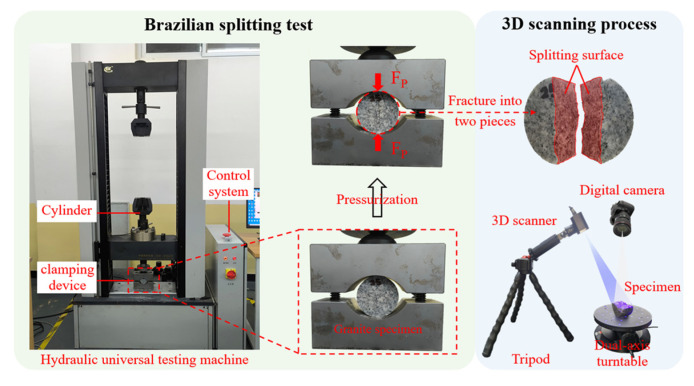
Brazilian splitting test and section scanning of the specimen.

Figure 6 illustrates the Brazilian splitting test apparatus and the specific 3D scanning procedure. The left image shows the hydraulic universal testing machine system, which comprises a hydraulic cylinder, a clamping device, and a closed-loop control system, designed for applying radially uniform pressure to the standard granite disc specimens. The loading rate was controlled at 0.5 MPa/s via a hydraulic system, which complies with the conventional loading rates recommended by the International Society for Rock Mechanics (ISRM) and relevant Chinese standards [36]. Upon reaching the critical load threshold, a specimen undergoes tensile fracture along its diameter, with cracks penetrating the entire cross-section, consequently resulting in its splitting into two pieces. This reflects the tensile failure characteristics of rock under biaxial stress conditions. The image on the right shows the 3D morphology acquisition system utilized in the experiment, which comprises a 3D scanner, a digital camera, and a dual-axis turntable. Through multi-angle optical scanning, the system precisely reconstructs the 3D morphology of splitting surfaces. The entire process exemplifies the analytical approach linking “macroscopic mechanical response” to “microscopic fracture morphology”. The high-precision 3D reconstruction enables the quantitative characterization of mesoscopic features associated with rock splitting failure, such as crack propagation paths and fracture surface roughness, thereby providing experimental evidence for the research of rock fracture mechanisms and strength theories.

## 3. Results and Discussion

### 3.1. Effects of Heating and Cooling Treatments

#### 3.1.1. Physical Properties

**Figure 7:**
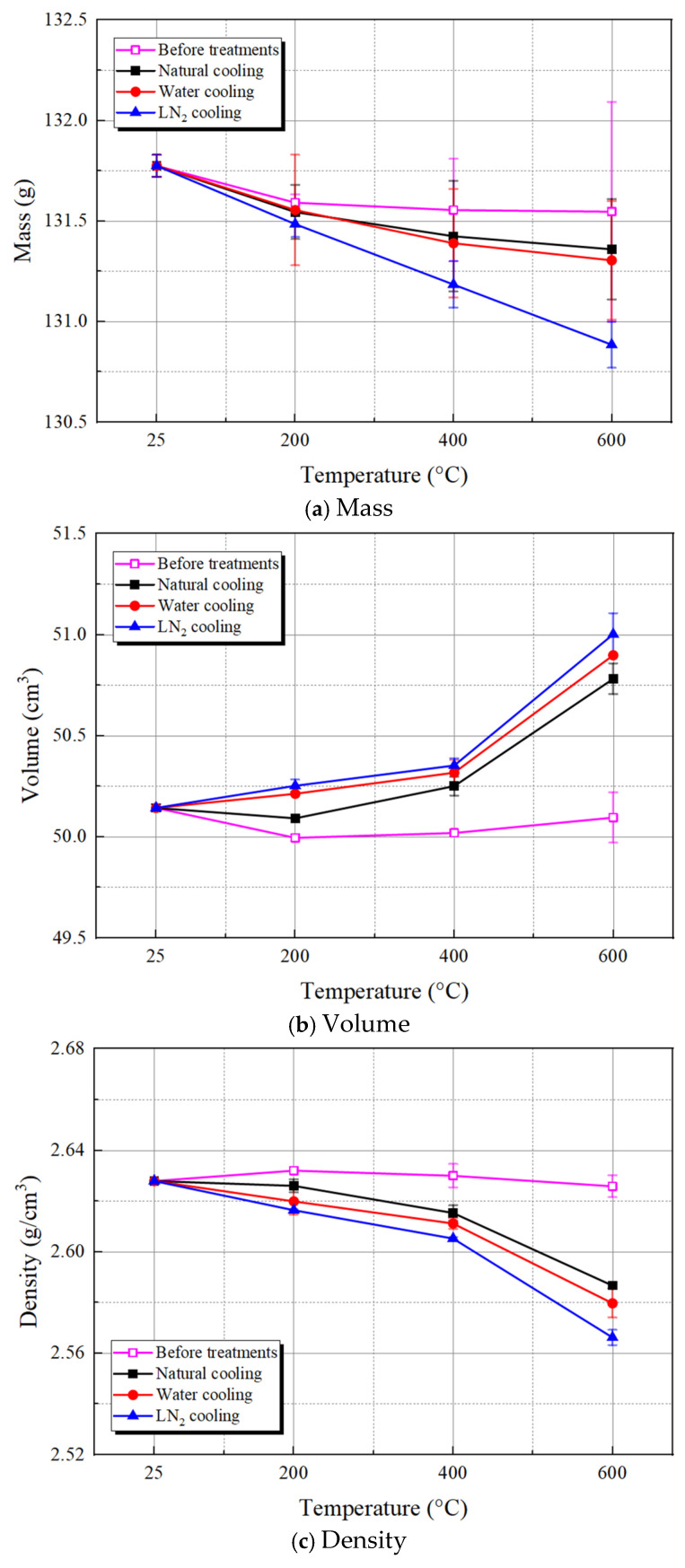

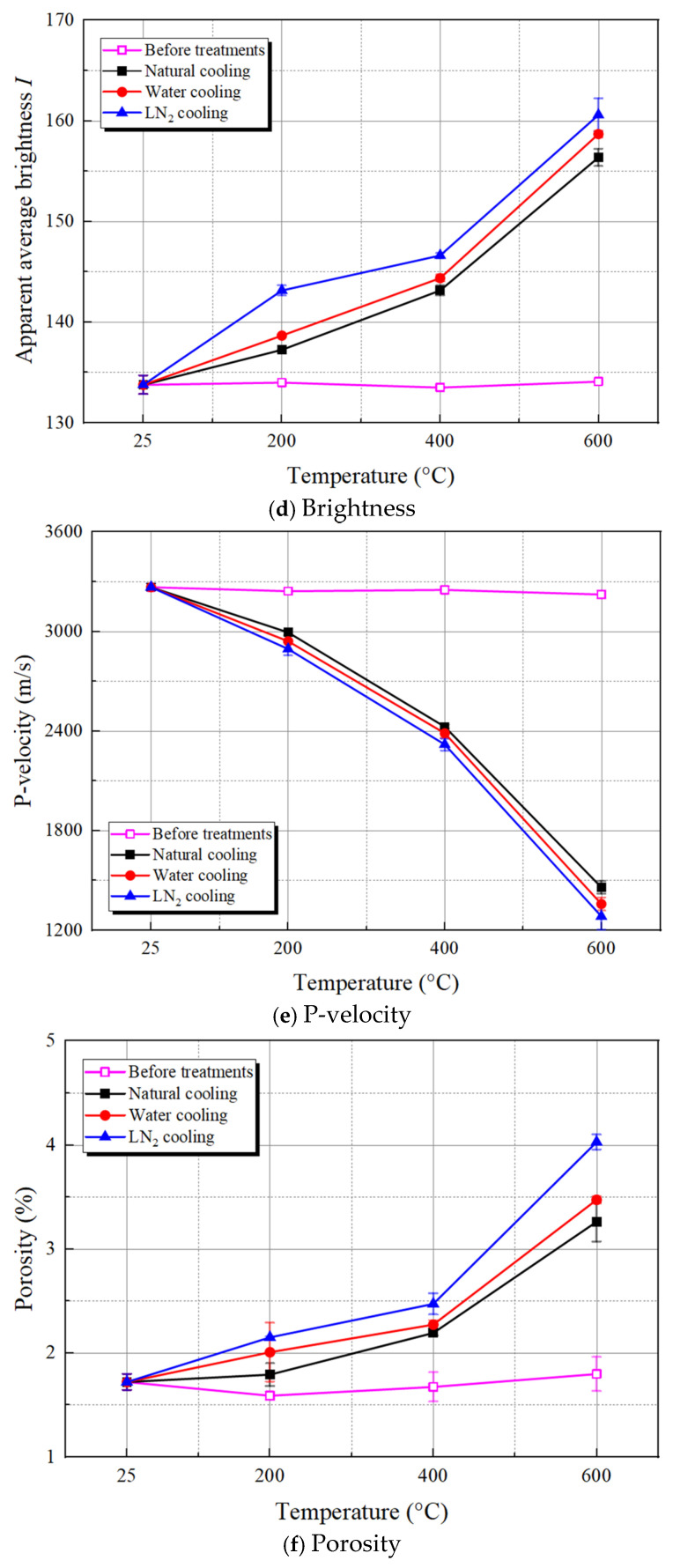
Physical properties of the thermal-treated granite with different cooling treatments.

Based on laboratory experiments, the basic physical properties (mass, volume, density, apparent brightness, P-wave velocity, and porosity) of thermal-treated granite were assessed, with specific results illustrated in Figure 7.

According to Figure 7a, the mass of granite decreases with rising temperature, and the cooling method demonstrates a significant path dependence on its mass loss, which becomes more pronounced after reaching 200 °C. When the temperature rises to 600 °C, the mass of granite decreases by 0.32%, 0.36%, and 0.68%, respectively, under the three cooling methods. Clearly, LN_2_ cooling caused the most severe mass loss, followed by water cooling, while natural cooling led to the least mass loss.

The volume alteration of granite specimens after heating and cooling treatments exhibits a significant, nonlinear, cumulative expansion pattern, as depicted in Figure 7b. Specifically, within the temperature range of 25 °C to 400 °C, the volumes of all three specimens increase slowly, with respective increments of 0.22%, 0.35%, and 0.42% for naturally cooled, water-cooled, and LN_2_-cooled specimens, compared to the room temperature result. This indicates the cumulative effect of thermal damage within the low to medium temperature spectrum. At a 600 °C temperature, the volumetric expansion of granite markedly intensifies, with volume increments rising to 1.27%, 1.51%, and 1.72%, respectively, when compared to the room temperature condition. This finding effectively validates the predominant role of the thermal-induced damage mechanism at high temperatures.

The density of granite, which is a key indicator describing the compactness of its internal structure, is closely linked to mechanical properties such as hardness and wear resistance [37]. As displayed in Figure 7c, under high temperature and cooling effects, the density of granite specimens initially experiences a slight decrease, followed by a rapid decrease, with the reduction becoming more pronounced after reaching 400 °C. When the temperature rises to 600 °C, the granite density is reduced by 1.57%, 1.84%, and 2.35%, respectively, under the three cooling treatments, relative to the room temperature condition. Evidently, LN_2_ cooling exerts the most substantial effect on the granite density, followed by water cooling, while natural cooling has the least effect.

The observed decrease in mass and density, along with the increase in volume of high-temperature granite, can be attributed to the differential thermal expansion of mineral grains inside the granite under high-temperature conditions, the initiation and propagation of microcracks, as well as the thermal shock effect generated during the cooling process [38]. Specifically, as the temperature rises, the lattice distortion and phase transitions in minerals such as quartz induce concentration of internal thermal stress, which facilitates the propagation of microcracks and exacerbates the cleavage and exfoliation between mineral grains, thereby causing mass loss and volumetric expansion of the specimens. LN_2_ cooling, characterized by its exceptionally large temperature gradient, introduces more drastic thermal shock inside the rock. Consequently, the microcrack propagation and spalling are accelerated, leading to significantly greater mass loss and volumetric expansion when compared to the water cooling and natural cooling scenarios. Conversely, the slow cooling associated with natural cooling allows for partial release and relaxation of thermal stress, which results in relatively smaller mass loss and volumetric expansion.

Figure 7d depicts the variation patterns of granite’s apparent average brightness I with temperature and cooling method. It can be observed that as the temperature rises, the apparent brightness values across three cooling methods all exhibit a monotonically increasing, nonlinear trend, with a higher cooling rate indicative of a greater increase in brightness. This phenomenon can be attributed to several factors: feldspar minerals (especially potassium feldspar) within the granite may undergo lattice distortion or partial melting at temperatures between 400–600 °C, causing alterations in their optical refractive index, which macroscopically manifests as a lightening or whitening of color. Meanwhile, the thermal expansion coefficients of different minerals vary significantly. During the heating process, the thermal stress generated between mineral grains thus induces the initiation and propagation of microcracks, leading to increased surface roughness of the grains and enhanced light scattering, which ultimately produces a “whitening” effect. Additionally, at high temperatures, slight oxidation reactions may occur on the mineral surfaces, resulting in the formation of a light-colored oxide film that further modifies the surface color [39].

As displayed in Figure 7e, the P-wave velocity of granite demonstrates notable degradation patterns and cooling media sensitivity in response to rising temperature and different cooling methods. At room temperature, the P-wave velocity of the rock specimens is approximately 3300 m/s. Following heating and cooling treatments, the velocity exhibits a gradual decline, reflecting the cumulative effect of thermal damage and the differentiated influences of cooling media on the microstructure. Specifically, at 200 °C, the P-wave velocities after natural, water, and LN_2_ cooling slowly decrease to 2995 m/s, 2942 m/s, and 2895 m/s, recording respective reductions of 8.34%, 9.96%, and 11.4%. When the temperature rises to 600 °C, the decline becomes more pronounced, with the velocities dropping to approximately 1459 m/s, 1359 m/s, and 1284 m/s, which correspond to reductions of up to 55.35%, 58.42%, and 60.7%, respectively. The decline in P-wave velocity of granite at high temperatures arises from a combination of mineral phase transitions and propagation of microcracks and micropores. Notably, the α-β phase transition of quartz occurring around 573 °C represents a critical threshold [40], making the 500–600 °C stage the most significant interval of P-wave velocity attenuation in granite. Meanwhile, rapid cooling intensifies the temperature gradient, triggering more pronounced thermal shock and uneven thermal stress. In particular, LN_2_ cooling leads to the most severe thermal cracking due to the extreme temperature gradient, thereby significantly diminishing the P-wave velocity of rock specimens.

Porosity is a crucial parameter for evaluating the internal damage of granite, reflecting its brittleness and strength. As displayed in Figure 7f, the porosity of granite increases slowly at first and then rapidly with rising temperature. Within the temperature range of 25 °C to 600 °C, the porosity increases from approximately 1.72% to about 3.26% after natural cooling, representing an increase of roughly 89.56%. Following subjection to water cooling, the porosity of granite increases from approximately 1.72% to about 3.47%, representing a roughly 101.79% increase. Under LN_2_ cooling, the porosity increases linearly from an initial value of around 1.72% to about 4.03%, which corresponds to an increase of 134.1%. Suggestively, severe thermal shock and phase transition effects exacerbate the deterioration of pore structure. When the temperature reaches 600 °C, the difference in porosity between water and natural cooling narrows to about 0.21%. This indicates that the accumulation of internal damage within the material gradually dominates the porosity evolution at high temperatures, thereby somewhat weakening the impact of differing cooling methods. The primary contributors to the increased porosity in granite under high-temperature conditions are the thermal stress induced by temperature gradient and the anisotropic expansion of mineral grains, leading to the formation of tensile cracks during the cooling process. Moreover, the higher the temperature, the greater the connectivity of microcracks, resulting in a more pronounced increase in porosity [41].

#### 3.1.2. Brazilian Test and Mechanical Properties

**Figure 8:**
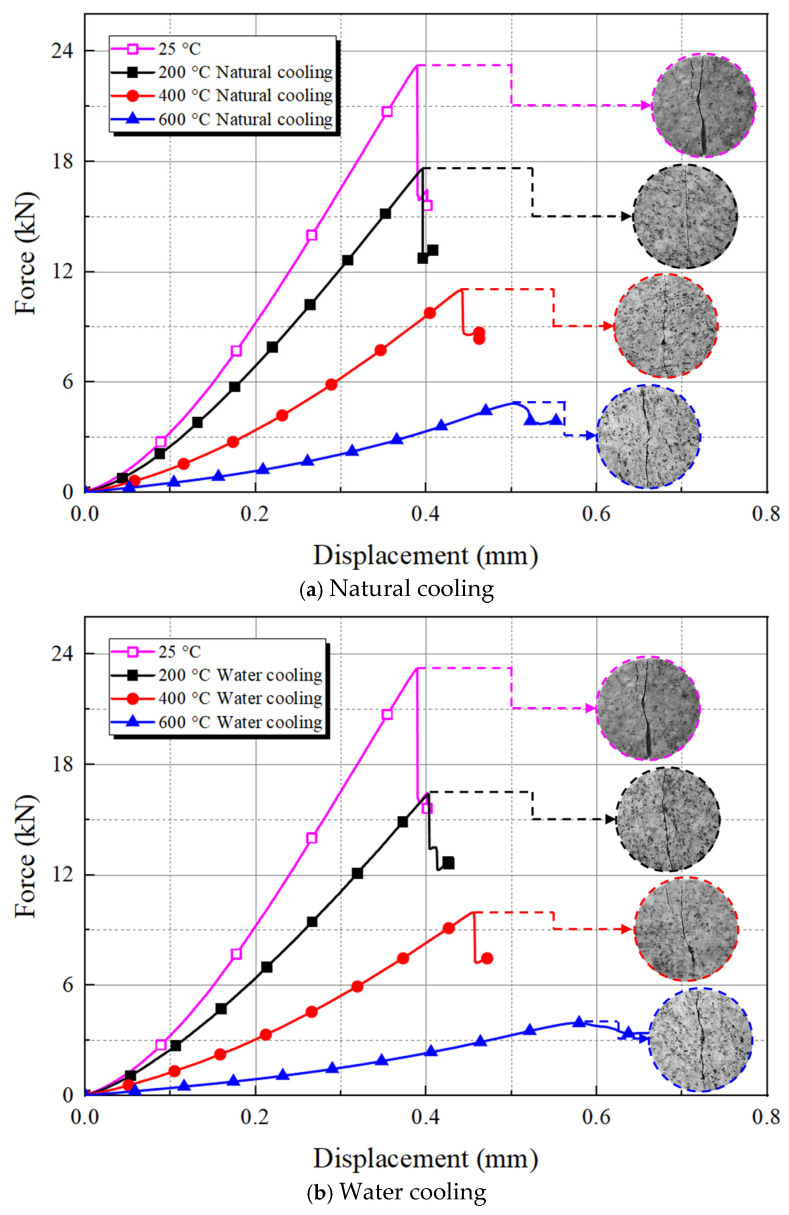

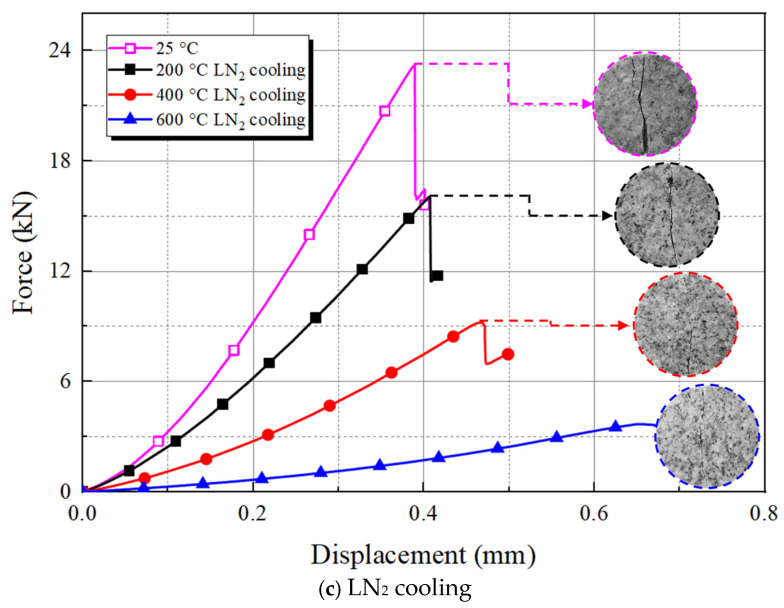
Load–displacement curves of the thermal-treated granite with different cooling treatments.

Brazilian splitting test was conducted on granite specimens that were subjected to different heating and cooling treatments, with the load–displacement curves illustrated in Figure 8. These curves of load–displacement evolution clearly reflect the failure process of granite treated at different heating temperatures and under various cooling conditions. The specimen curves exhibit similar trends across different heating temperatures and cooling methods. They consistently show a concave-upward trend during the initial compaction stage of load application, then transition into the elastic stage and exhibit a linear pattern. As the external force increases continuously, all specimens experience a sudden drop in load upon reaching the peak load. During the descending stage following peak load, all curves reveal a distinct serrated pattern, indicating that all specimens demonstrate obvious characteristics of brittle failure [42].

At a relatively low heating temperature (200 °C), the dehydration process (involving loss of adsorbed, zeolitic, and weakly bound water), along with the thermal cracking phenomenon, causes the formation of a few cracks and pores, which somewhat prolong the compaction stage of the load–displacement curves. Nevertheless, the peak load does not exhibit a significant decrease due to the closure of some cracks that arise from mineral expansion. As the temperature further rises (400 °C), the accumulation of damage leads to a further decrease in the load curve slope and a substantial decline in peak load. Upon reaching 600 °C, the escape of crystallization water inflicts damage on the mineral lattice structure, resulting in enlarged internal defects and easier connectivity. Moreover, after surpassing 573 °C, the quartz inside the granite undergoes a phase transition, which further weakens the bonding between mineral grains, consequently leading to a considerable prolongation of the compaction stage, a shortening of the elastic stage, and a decline in peak load on the load–displacement curves.

Additionally, at the peak load points of the curves in Figure 8, the failure patterns of different thermal-treated granite specimens after undergoing the Brazilian splitting test are presented. Comparative analysis indicates that no significant change occurs in the Brazilian splitting failure mode of the specimens under different conditions, and the resulting macroscopic cracks are generally found on the plane where the loading points at both specimen ends are located. The fracture surfaces of some specimens are not regular planes, with slight bending appearing at both ends of the splitting cracks, and certain tortuosity being noted at the crack centers. The reason is that the internal damage caused by heating granite enhances its strength anisotropy. During the splitting process, there is a tendency for sliding along faces with a weaker granular bonding force, where the specimens experience not only primary tensile failure but also some shear failure. As the temperature rises, the number of granules detached around the fracture surface due to splitting also increases, and the fracture surface gradually transforms from being dense and smooth to rough and fragmented. Nonetheless, the specific quantification and comparison of the section roughness patterns for granite treated with high-temperature heating and cooling require further research.

By analyzing the load–displacement curves of granite, the variation laws of mechanical parameters, such as the maximum radial displacement and splitting tensile strength in the Brazilian test, can be derived for the rock specimens [42]. The tensile strength is determined using the following formula:

(1) $$\sigma (t)=\frac{2P(t)}{\pi DB}$$

where *σ*(*t*) signifies the tensile strength, MPa. *P*(*t*) indicates the axial peak load, kN. *D* and *B* denote the diameter and breadth of the granite disc specimen, respectively, in mm.

**Figure 9:**
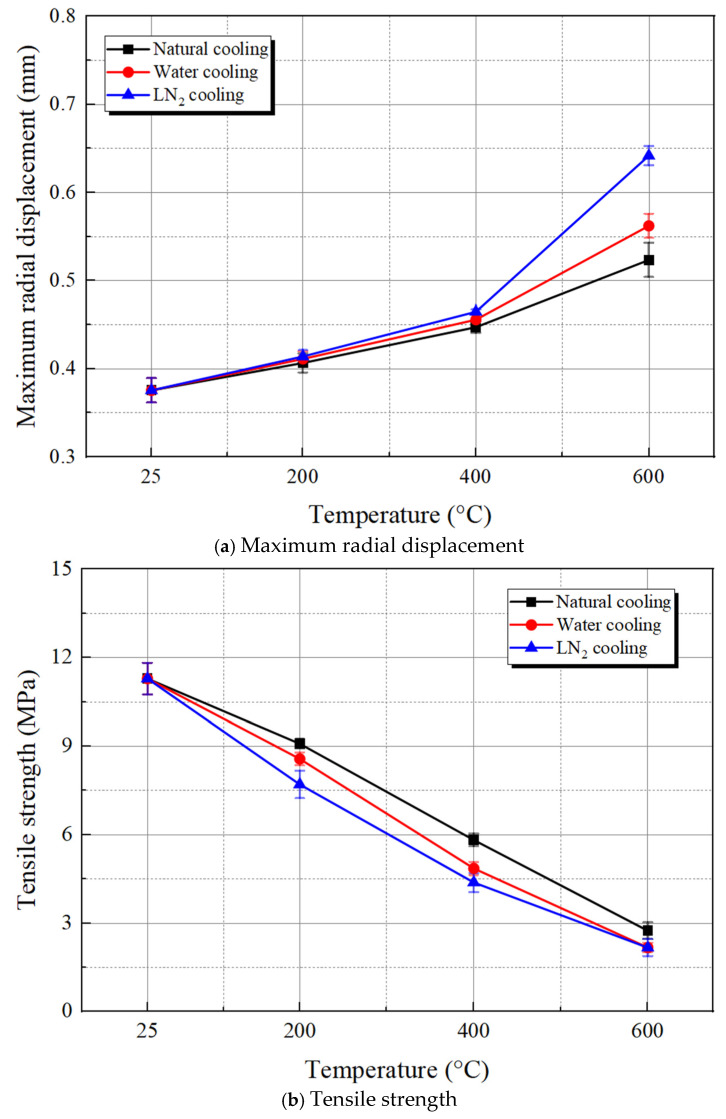
Mechanical properties of the thermal-treated granite with different cooling treatments.

Figure 9a depicts the maximum radial displacement recorded in the Brazilian splitting test for specimens exposed to different temperatures and cooling conditions. Clearly, high-temperature heating followed by rapid cooling leads to a certain staged nature of the final failure deformation for the specimens. When the temperature is below 400 °C, the rise in temperature causes an expansion of compressible space and a reduction in specimen brittleness, which in turn enables a continuous increase in its deformation under external force. As the temperature further rises, the diminished mineral bonding strength results in specimen failure under lower loads, while the increasing plasticity of the specimens leads to a continued increase in their maximum radial displacement. Comparing the three cooling methods at identical heating temperatures, LN_2_ cooling significantly influences the specimen deformation characteristics more than water cooling and natural cooling, causing noticeable deformation even under lower load conditions.

The splitting tensile strength calculations for the granite specimens under various conditions are illustrated in Figure 9b. The tensile strength of these specimens also exhibits a notable correlation with their internal damage state. Unlike the variation pattern of maximum radial displacement, the tensile strength consistently decreases with rising temperature. Compared to the room temperature condition, the average tensile strength of 600 °C granite treated with three cooling methods decreases by 75.68%, 80.74%, and 80.79%, respectively. A comparative analysis of the test results reveals that the granite’s tensile strength is significantly more sensitive to temperature than to the cooling method. Moreover, at the same temperature, granite cooled with LN_2_ has the lowest tensile strength, followed by that subjected to water cooling, while natural cooling yields higher tensile strength. This finding is attributed to the thermal expansion of minerals within granite at high temperatures, the propagation of microcracks, as well as the severity of thermal shock associated with various cooling methods. High temperatures induce thermal stress within granite due to the differing thermal expansion coefficients of its mineral grains, leading to the initiation and propagation of microcracks, which ultimately diminish the rock’s tensile strength. LN_2_ cooling, characterized by its extremely high cooling rate, induces a more severe thermal shock, which further exacerbates the propagation of microcracks within granite, thereby yielding a lower tensile strength compared to water cooling and natural cooling.

### 3.2. Thermal Damage

#### 3.2.1. Thermal Damage Versus Temperature

Through the analysis of rock’s splitting tensile strength and failure mode, we find that various heating and cooling treatments have non-negligible effects on the mechanisms and processes associated with rock tensile failure. These treatments may induce severe thermal shock damage to the rock specimens, consequently degrading their physical properties. To quantitatively describe the effects of these treatments on the granite specimens prior to the impact test, the concept of thermal damage degree was introduced.

According to previous research, the P-wave velocity in rock is intricately linked to the degree of rock damage, with a greater variation in P-wave velocity indicating more pronounced rock damage. Hence, a thermal damage value related to the P-wave velocity was defined to measure the degree of rock damage [43], as formulated in Equation (2):

(2) $$D_{V}(T)=1-\frac{c_{1}^{2}}{c_{0}^{2}}$$

where *D_V_* signifies the thermal damage factor. *c*_0_ and *c*_1_ denote the P-wave velocities of rock before and after the heating and cooling treatments, respectively, m/s.

**Figure 10:**
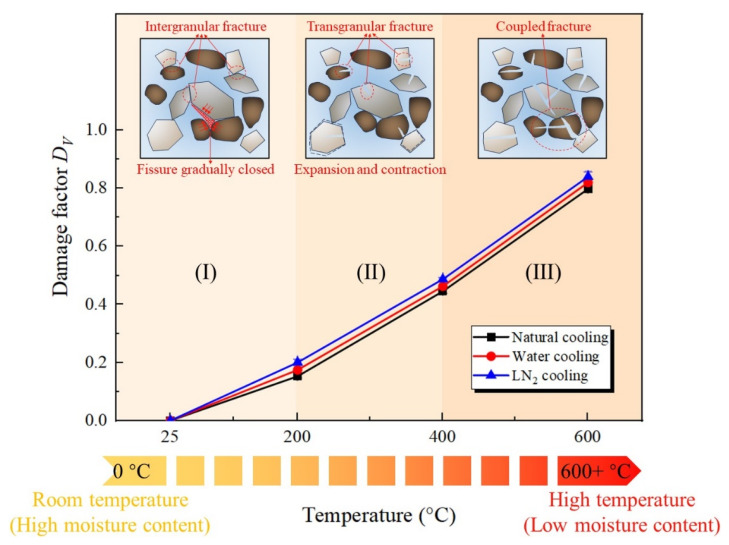
Damage factor of thermal-treated granite based on P-wave velocity.

As displayed in Figure 10, the thermal damage factor *D_V_* of granite exhibits a significant monotonically increasing trend with rising temperature, while also demonstrating a distinct staged nature and dependence on the cooling method. Combining previous research conclusions, it is evident that during temperature increases, the moisture content inside the rock progressively decreases and uneven expansion occurs due to the differing thermal expansion coefficients of mineral grains. When the thermal stress on these mineral grains surpasses a certain threshold, microcracks develop inside the rock [44,45]. Meanwhile, upon reaching a specific temperature level, minerals undergo phase transitions and decomposition, thereby further exacerbating the internal damage of the rock. The figure illustrates that the progression of damage factor increase can be divided into three distinct stages:

(1). Within the temperature range of 25 °C to 200 °C, the damage factors of granite treated with three cooling methods increase slowly from 0 to 0.15, 0.17, and 0.20, respectively. The damage evolution is relatively mild, which can be attributed to the initial predominance of intergranular fracture among the rock grains. Consequently, this leads to the closure of internal microcracks and the evaporation of pore water, with the thermal stress effect remaining insignificant.
(2). In the 200–400 °C range, the damage factor increases markedly compared to the 200 °C scenario, indicating that the generation of thermal stress results in a transition of internal rock damage from being predominantly intergranular to predominantly transgranular. Meanwhile, the disparities in thermal expansion and contraction within the rock contribute to the exacerbation of fracture network formation.
(3). During the high-temperature stage of 400 °C to 600 °C, the damage factor of granite continues to increase at a high rate, with respective values reaching 0.80, 0.82, and 0.84 under the three cooling treatments. This observation is closely linked to the substantial thermal stress caused by the variations in thermal expansion coefficients of mineral grains at high temperatures, resulting in coupled intergranular and transgranular damage.

Furthermore, comparison of the three cooling methods reveals that the damage factors under water and LN_2_ cooling are consistently slightly greater than those observed with natural cooling. In particular, within the temperature range above 400 °C, the thermal shock effect induced by LN_2_ cooling is the most pronounced. The damage factor for 600 °C granite specimens is approximately 5.25% greater than that of naturally cooled specimens, indicating that under rapid cooling conditions, the surface of high-temperature granite cools faster while its interior cools more slowly. Given this substantial temperature difference between the specimen’s interior and surface, considerable thermal stress is generated, which exacerbates the internal structure deterioration. This pattern, encompassing the microcrack initiation to the thermal stress-driven crack propagation and culminating in a complete penetrative failure, profoundly reflects the thermal damage mechanisms of granite subjected to high temperature and varying cooling rates. This finding provides crucial experimental evidence for the stability evaluation and cooling strategy development associated with high-temperature rock masses in deep engineering contexts.

#### 3.2.2. Thermal Damage Versus Tensile Strength

**Figure 11:**
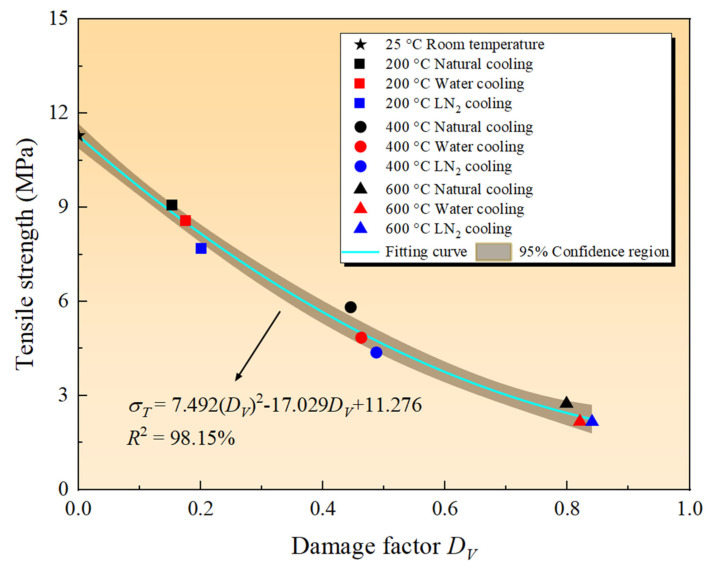
Relationship between damage factor *D_V_* and tensile strength.

During the heating and cooling processes of granite specimens, both the tensile strength and internal damage of the specimens fluctuate in response to temperature rises and variations in cooling methods, rendering it meaningful to reflect the specimen strength level through internal damage. Accordingly, damage factor *D_V_* was employed as an indicator to quantify the macroscopic strength variations in the specimens [46]. The *D_V_* values for granite specimens subjected to various conditions are matched one-to-one with their tensile strengths, as plotted in Figure 11. Drawing from previous experience, it is posited that the tensile strengths of specimens subjected to different heating treatments follow a quadratic polynomial relationship with the damage factor. After fitting the specimen tensile strength σ*_T_* and the internal damage factor *D_V_*, we derived the following equation. The coefficient of determination R_2_ for the fitted curve is 0.98, indicating a good correlation between the two variables.

(3) $$\sigma_{T}=7.492{\left(D_{V}\right)}^{2}-17.029D_{V}+11.276$$

The figure indicates that as the thermal shock damage increases gradually, the tensile strength of granite decreases progressively. Based on the fitted curve characteristics, when the thermal shock damage *D_V_* increases, the trend of granite’s tensile strength *σ_T_* decline changes from rapid to slow. At *D_V_* values less than 0.4, the *σ_T_* value of granite decreases by approximately 49.83% compared to that of the rock at room temperature. When *D_V_* ranges between 0.4 and 1.0, the *σ_T_* value of granite exhibits a slow decline.

This variation pattern arises because thermal shock damage is a key indicator affecting the granite’s strength. At high temperatures, differential thermal expansion occurs among the mineral grains within the granite, leading to the initiation and propagation of microcracks. The subsequent cooling processes (especially water and LN_2_ cooling) intensify the thermal shock effect, causing further penetration of the microcracks, which macroscopically manifests as a continuous reduction in tensile strength. Noticeably, at equivalent levels of damage, the difference in the effects of various cooling treatments on strength is minimal, suggesting that the dominant role played by thermal damage in the granite tensile strength arises from the effect of high temperature on the rock properties, while the impact of cooling method is relatively minor.

### 3.3. Failure Mode and Roughness of Fracture Surface

#### 3.3.1. Different Roughness Definition Methods

**Figure 12:**
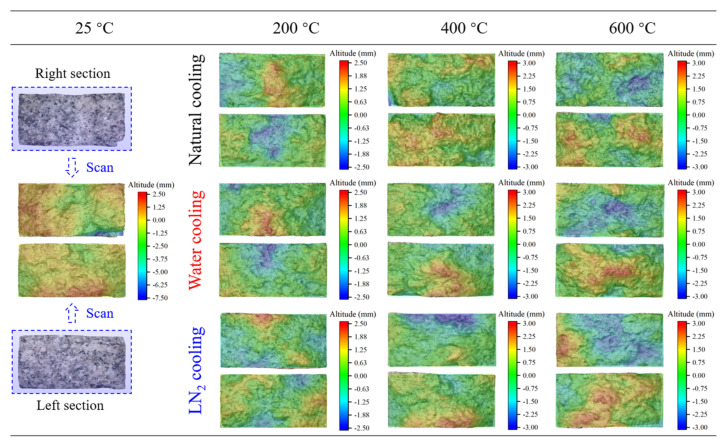
Effect of different cooling treatments on fracture surface morphology of granite.

In Figure 8, the tensile failure mechanism of thermal-treated granite is described based on load–displacement curves. Evidently, the splitting cracks in all disc specimens are located at the specimen center and aligned parallel to the loading direction. To better elucidate the relationship between the macroscopic mechanical response of high-temperature granite and the microscopic fracture morphology, and to precisely analyze the effects of various cooling treatments on failure mode, a Revopoint MINI 3D scanner system (Revopoint, Xi’an, China) was utilized to examine the morphology of the fracture surface and assess its roughness [47]. Figure 12 illustrates the fracture surface morphology of granite after undergoing various cooling treatments, reconstructed through 3D scanning. Different colors in the figure indicate the heights of measurement points.

**Figure 13:**
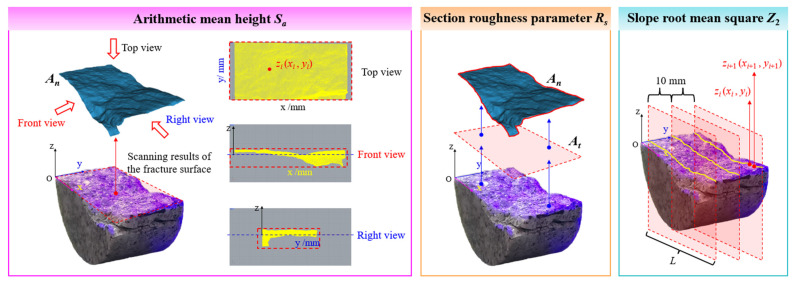
Different types of section roughness calculation methods.

Building upon previous research, we employed three common parameters, namely arithmetic mean height *S_a_*, section roughness parameter *R_S_*, and slope root mean square *Z*_2_, to evaluate the fracture surface roughness of granite under different conditions [48,49,50], with a view to identifying the most appropriate definition method through comparative analysis. Figure 13 systematically illustrates the definitions and computational principles of these three roughness parameters, which serve to quantify the microscopic morphological features of 3D fracture surfaces. Among them, the arithmetic mean height *S_a_* characterizes the macroscopic undulation of a surface by integrating and averaging the absolute height deviations of all discrete points within the entire sampling area relative to a reference plane. The section roughness parameter *R_S_* reflects the unevenness of a fracture surface by calculating the ratio of actual surface area intercepted by the section to its projected area. Meanwhile, the slope root mean square *Z*_2_ emphasizes the variations in surface height gradient across granite fracture surfaces. From the center of each specimen’s splitting surface, a computational profile was extracted at 10 mm intervals. By statistically analyzing the root mean square of the ratio between height difference and horizontal distance of adjacent sampling points on the computational profile, we quantitatively described the local inclination of the surface and the steepness of the microscopic structure. The three parameters collectively establish a multi-scale characterization system for the geometric roughness of complex fracture surfaces from three perspectives–global height distribution, sectional unevenness, and structural inclination, providing a vital quantitative foundation for a deeper understanding of material fracture mechanisms and surface functional properties.

The computational formulas for the three parameters are as follows:

(4) $$\left\{\begin{array}{l} S_{a}=\frac{1}{A_{t}}\int_{A_{t}}\left|z_{i}\left(x_{i},y_{i}\right)\right|dxdy\\ R_{S}=A_{t}/A_{n}\\ Z_{2}={\left[\frac{1}{L}\sum \frac{{\left(z_{i+1}-z_{i}\right)}^{2}}{x_{i+1}-x_{i}}\right]}^{\frac{1}{2}} \end{array}\right.$$

where *A_t_* represents the actual area of scanned section, mm^2^. *A_n_* is its projected area on the reference plane, mm^2^. *z_i_*(*x*, *y*) denotes the height of a point on measured surface; *x_i_* and *y_i_* indicate the horizontal and vertical coordinates of measured point; *x_i_*_+1_ − *x*_i_ signifies the distance between the horizontal coordinates of two measured points on the computational profile; *L* denotes the maximum length of selected computational profile, mm.

**Figure 14:**
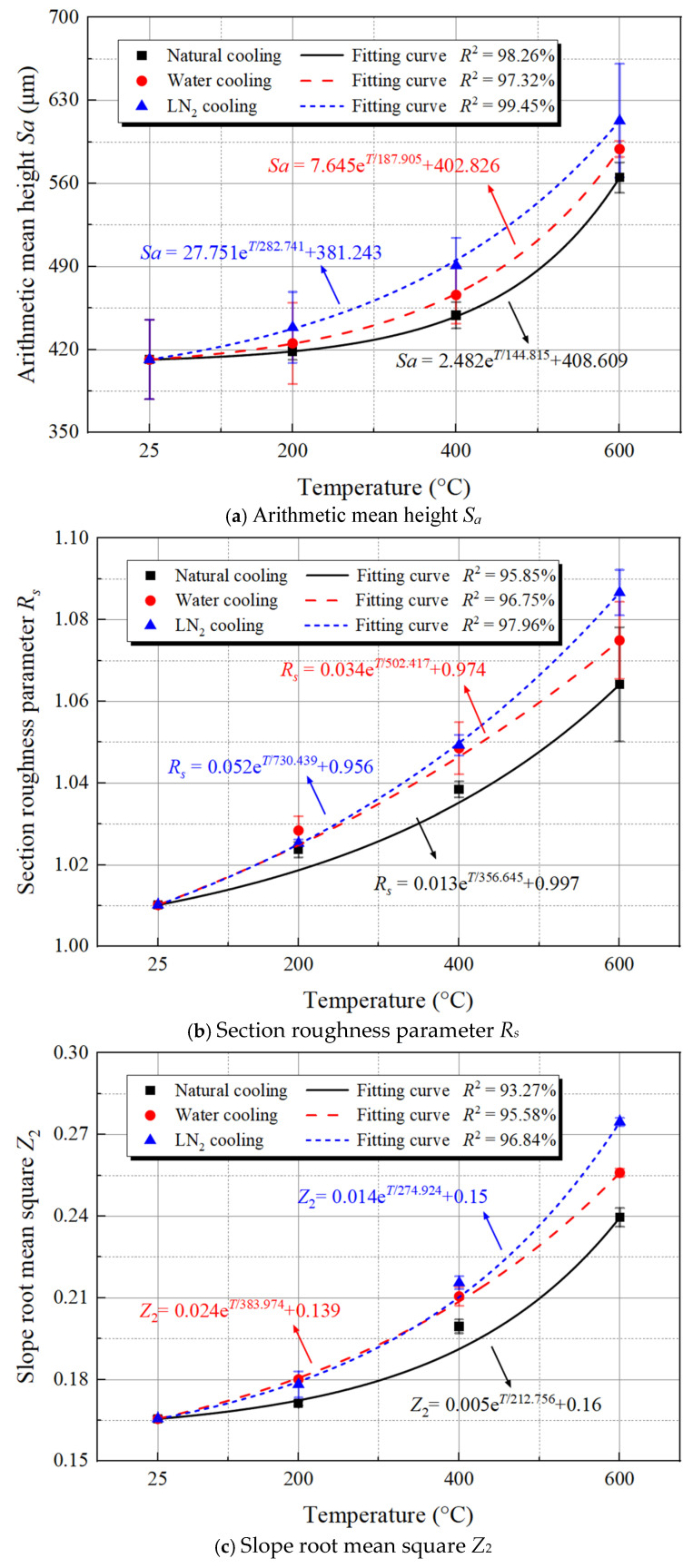
The variation in three kinds of roughness parameters with temperature.

Computations were performed based on Equations (4) and the three roughness characterization methods described in Figure 13, with the results plotted in Figure 14. This figure systematically reveals the nonlinear evolution patterns of *S_a_*, *R_S_*, and *Z*_2_ on the surface of thermal-treated granite exposed to three distinct cooling treatments. All three parameters exhibit a monotonically increasing, exponential growth trend with rising temperature, with the growth rates significantly accelerating after reaching 400 °C. Comparison of various cooling conditions reveals that the increase in parameters is greatest under LN_2_ cooling, followed by water cooling, while the smallest increase is observed under natural cooling. This observation is primarily attributed to the severe thermal shock caused by LN_2_ cooling, which leads to greater residual stress and microstructural distortion within the material, thereby amplifying the dynamics of surface roughening. Conversely, natural cooling effectively mitigates the initiation and propagation of such defects owing to its gentle temperature gradient.

The three kinds of roughness parameters demonstrate similar nonlinear exponential growth trends with temperature, suggesting that the evolution of material surface roughness is a temperature-driven systematic process that integrates multi-scale features. These parameters characterize the surface morphology from different perspectives, and their concurrent growth indicates that the rise in temperature holistically drives the disordering and roughening of surface microstructure via thermal activation mechanisms (e.g., atomic diffusion, lattice distortion, phase transition, or oxidation), rather than merely affecting the surface features at a specific scale. This consistency reveals that temperature, as a core thermodynamic driver, can uniformly regulate the multi-scale morphological evolution of material surfaces from microscopic roughness to macroscopic contour. Its underlying mechanism may be intricately linked to the internal defect proliferation, grain enlargement, or uneven development of surface oxide layers in the material at high temperatures. The disparities in cooling methods (with the highest increase observed in LN_2_ cooling and the lowest in natural cooling) further corroborate that the temperature gradient and cooling rate, by affecting the thermal stress distribution and phase transition dynamics, concurrently act on the evolutionary paths of all roughness parameters, ultimately resulting in similar exponential growth patterns of the three parameters under different cooling conditions. Nevertheless, the specific magnitude of growth varies due to the microstructural differences caused by the cooling methods. Suggestively, the temperature dependence of surface roughness is essentially a macroscopic reflection of the material’s microstructural evolution under coupled thermo-mechanical effects. Its characteristic of multi-parameter synergistic variation offers a theoretical basis for the multi-scale evaluation and regulation of material surface integrity in extreme temperature environments.

#### 3.3.2. Relationship Between Roughness and Mechanical Characteristics

**Figure 15:**
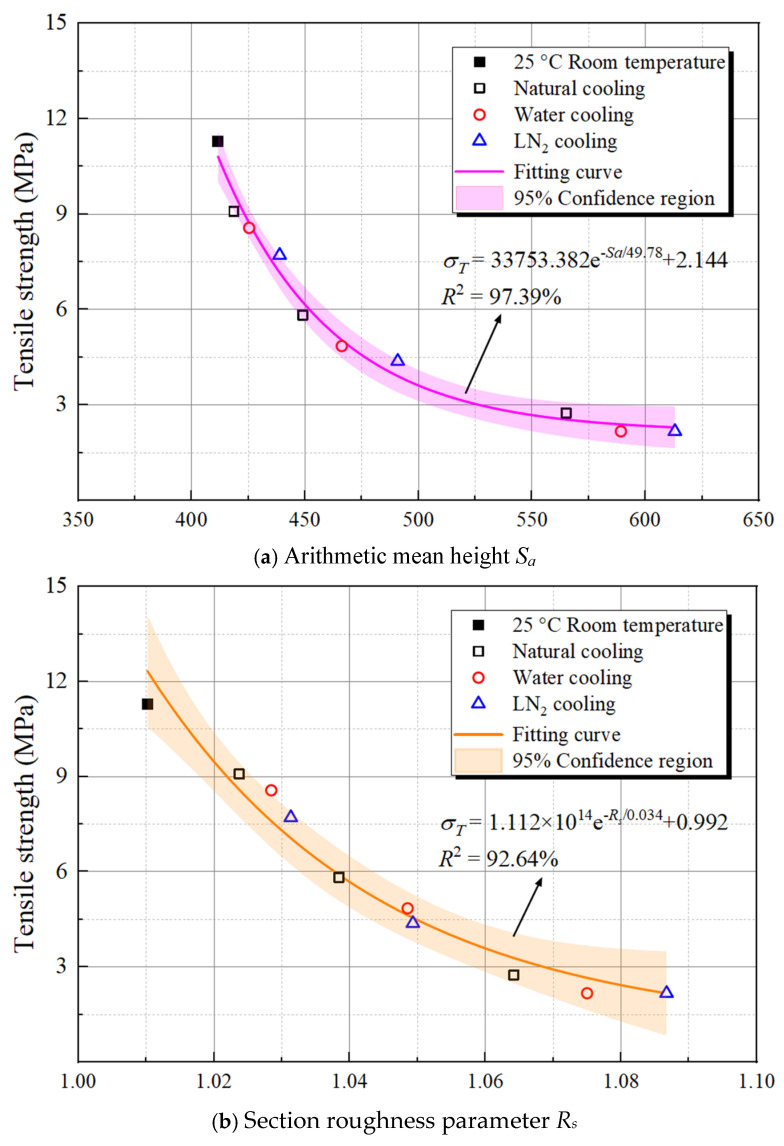

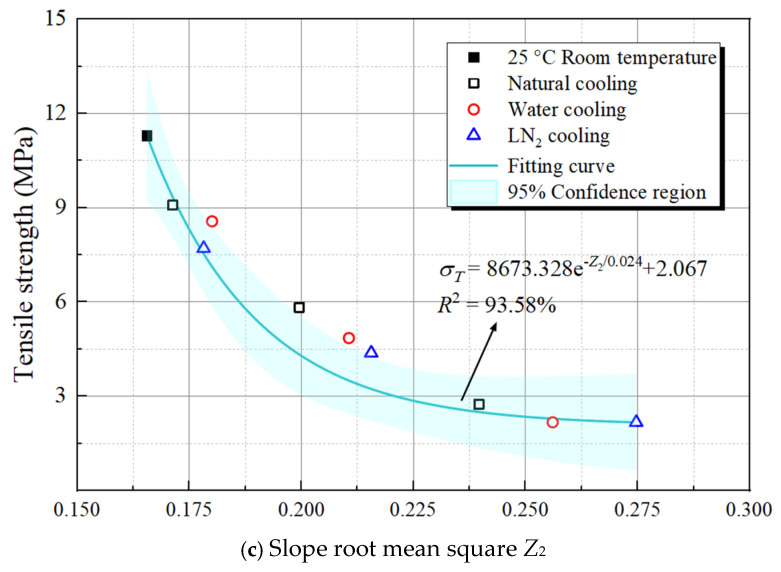
The relationship between section roughness and tensile strength of thermal-treated granite.

In materials science and engineering applications, surface roughness directly influences the stress concentration, interface bonding, and fatigue properties of materials. As the core means of regulating microstructure and surface state, the effect of the cooling process on the final mechanical properties of materials necessitates urgent clarification. Figure 15 systematically illustrates the quantitative relationship between the section roughness parameters of granite material and its tensile strength across different cooling methods, unveiling the key mechanisms that influence the material’s mechanical properties. The three subgraphs in the figure respectively show the nonlinear decay patterns of tensile strength in relation to the arithmetic mean height *S_a_*, section roughness parameter *R_S_*, and slope root mean square *Z*_2_.

In this figure, an identical type of fitted curves is utilized to separately model the correlations of roughness indicators with tensile strength. The shaded area represents the 95% confidence interval of the fit, which characterizes the data dispersion. The goodness of fit R^2^ for all three fitted curves exceeds 92%, indicating highly significant correlations between the roughness indicators and tensile strength. Moreover, the variations in goodness of fit and confidence intervals among the three indicators, after the quantification process, demonstrate that these roughness parameters possess differing capabilities in characterizing the material tensile strength. Specifically, the goodness of fit is highest when *S_a_* is used as the variable (R^2^ = 97.39%), followed by that using *Z*_2_ (R^2^ = 93.58%), while *R_S_* corresponds to the lowest goodness of fit (R^2^ = 92.64%). This disparity indicates that *S_a_*, as a comprehensive parameter characterizing the fluctuations of material section height, more fully represents the overall features of surface morphology. It is more closely linked to the microstructural evolution and stress concentration effect inside the material that arises from the cooling process, thereby having stronger explanatory power for the influence pattern on tensile strength. In contrast, *R_S_* primarily describes the sectional features of surface contour, which provides relatively limited dimensions of information, making it somewhat less effective in characterizing the correlation between complex surface morphology and mechanical properties. While *Z*_2_ can indicate the variation trend of surface height difference, its sensitivity to surface microscopic details is slightly inferior to *S_a_*, placing its goodness of fit between the other two indicators.

As the three roughness indicators *S_a_*, *R_S_*, and *Z*_2_ increase, the material’s tensile strength exhibits an exponential decay trend. This observation is attributed to the increase in surface roughness, which intensifies the stress concentration effect, increases the number of microdefects, and enhances the driving force for crack initiation and propagation, thereby considerably weakening the material’s load-bearing capacity. Meanwhile, the differences in test data point distribution across various cooling methods also reflect the synergistic regulatory effect of cooling rate on the surface morphology evolution and mechanical properties, providing both experimental evidence and theoretical support for a deeper understanding of multi-failure mechanisms in high-temperature granite from deep geological formations.

## 4. Conclusions

Experiments on the thermal damage mechanisms and mechanical properties of thermal-treated (200 °C, 400 °C, and 600 °C) granite subjected to various cooling methods (natural cooling, water cooling, LN_2_ cooling) have been conducted. A combination of XRD and FE-SEM was employed to analyze the mineral composition and microstructure of the granite. Furthermore, by applying 3D scanning technology, fracture surfaces were reconstructed, three parameters for evaluating fracture surface roughness were defined, and a quantitative model correlating thermal damage factor with tensile strength was developed. The major conclusions are drawn as follows:

(1) As the temperature rises (25–600 °C), the mass of granite exhibits a decreasing trend, while its volume and porosity increase nonlinearly. Notably, at temperatures above 400 °C, the rates of volumetric expansion and porosity growth increase significantly due to the intensified differences in thermal expansion among mineral grains, as well as the penetration of internal microcracks. Moreover, the physical parameters of granite demonstrate a considerable path dependency on the cooling method. LN_2_ cooling yields the most severe thermal shock, resulting in the greatest mass loss and volumetric expansion (a 2.35% reduction in density at 600 °C). This observation confirms that rapid cooling exacerbates the loosening of granite’s internal structure.

(2) With the elevation in temperature, the failure mode of rock transitions from typical brittle fracture to ductile failure. The load–displacement curve indicates that at 600 °C, the rock compaction stage is markedly prolonged, and the peak strength is substantially reduced. Tensile strength decreases exponentially with rising temperature. At temperatures below 600 °C, the tensile strength of LN_2_-cooled granite decreases by 80.79% compared to the room temperature value. This reduction is greater than that observed with water cooling and natural cooling. The tensile strength of granite is significantly more sensitive to temperature than to cooling method, with high temperature-induced mineral lattice distortion and phase transition constituting the intrinsic contributors to strength degradation.

(3) The thermal damage factor *D_V_* increases progressively in three stages with rising temperature. In the 400–600 °C stage, the *D_V_* value surges from approximately 0.4 to 0.84 (LN_2_ cooling), thereby confirming a damage mechanism predominantly characterized by coupled intergranular-transgranular fracture at high temperatures. A quadratic polynomial model correlating the thermal damage factor with the tensile strength is developed, revealing the dynamic process of strength decay from rapid to slow as the thermal damage intensifies. This model serves as a theoretical framework for evaluating the stability of hot dry rock reservoirs under thermal stress effects.

(4) The arithmetic mean height *S_a_*, section roughness parameter *R_S_*, and slope root mean square *Z*_2_ all exhibit a temperature-dependent trend of exponential growth. At 600 °C, the fracture surface of granite transforms from relatively dense and smooth to highly rough and fragmented, with the roughening effect being the most pronounced under LN_2_ cooling conditions. Furthermore, by establishing a relationship between roughness parameters and tensile strength, we verify that the arithmetic mean height *S_a_* is the most effective parameter for characterizing rock strength (goodness of fit *R*^2^ = 97.39%). Suggestively, high-temperature thermal shock alters the microscopic fracture mechanism of granite, which intensifies the fracture surface undulation, consequently resulting in a loss of macroscopic bearing capacity.

## Data Availability

The original contributions presented in this study are included in the article. Further inquiries can be directed to the corresponding author.

## Abbreviations

The following abbreviations are used in this manuscript:

XRD	X-ray diffraction
FE-SEM	Field emission scanning electron microscopy
LN_2_	Liquid nitrogen
3D	Three-dimensional
AE	Acoustic emission
DIC	Digital image correlation
NMR	Nuclear magnetic resonance
ISRM	International Society for Rock Mechanics
